# Mapping the evidence of self-compassion in caregiver wellbeing for caregivers of persons with neurodegenerative disease: A scoping review

**DOI:** 10.1017/S1478951524001639

**Published:** 2025-01-21

**Authors:** Christi M. Lero, Soobin Park, Emily L. Mroz

**Affiliations:** 1 Brown School of Social Work, Washington University in St. Louis, St. Louis, MO, USA; 2 Section of Geriatrics, Department of Internal Medicine, Yale School of Medicine, New Haven, CT, USA

**Keywords:** Caregiver, neurodegenerative disease, self-compassion, wellbeing

## Abstract

**Objectives:**

Caregivers of those with neurodegenerative disease (ND) manage complex symptoms which impact their wellbeing. Self-compassion can promote maintenance of wellbeing during challenging experiences, including caregiving. Little guidance exists for observationally studying self-compassion or targeted interventions for this population. Our objective was to complete a scoping review of research describing self-compassion in the context of caregiver wellbeing of caregivers of those living with ND.

**Methods:**

Following Preferred Reporting Items for Systematic Reviews and Meta-analysis extension for Scoping Reviews (PRISMA-ScR) guidelines, 3 online databases identified 350 peer-reviewed articles, 18 of which were included in this study. Eligibility included being written in English, targeting caregivers of those living with ND, and examination of self-compassion. Articles were organized by the incorporation or characterization of self-compassion in the study design.

**Results:**

Alzheimer’s disease predominated study samples of care recipients. Across study types self-compassion appeared as a theoretical concept, emerging theme, variable associated with other outcomes, and main outcome variable. Self-compassion is frequently measured using the Self-Compassion Scale, full or short form .

**Significance of results:**

The study of self-compassion with caregivers of individuals living with ND is growing. Current literature is somewhat unfocussed, leading to gaps in understanding conceptualization to achieve maximum intervention benefits. Clarifying the role of self-compassion in caregiver wellbeing will provide a lens through which non-pharmacologic, psychotherapeutic, and behavioral intervention development may be framed to reduce negative psychological outcomes. The most frequently represented ND is Alzheimer’s disease or other dementia, obscuring other NDs like amyotrophic lateral sclerosis, Parkinson’s disease, and others.

## Introduction

Neurodegenerative disease (ND) associated with a steady loss of neurons in the brain and body, leading to severe decline and loss of function, often over several years (Lamptey et al., 2022). Due to the significant loss of function, persons living with ND (PWND) come to rely heavily on caregivers, including family members, friends, or others, who provide support for activities of daily living. The growing aging population in the United States brings an increased prevalence of ND (Hou et al. 2019), and as a consequence, caregivers have been increasingly called to support these individuals. Caregivers of PWND manage progressively complex and prolonged care in service of wellbeing for the care recipient given that each ND is progressive, incurable, and irreversible (Aoun et al. 2013; Brizzi et al. 2020; Connolly et al. 2015; Galvin et al. 2018; Poppe et al. 2020). As caregivers channel extensive energy to these care activities, they also experience threats to their own health; most notably, these caregivers report extreme stress that lead to breakdowns in their functioning in their own lives (Han et al. 2022; Sommers-Spijkerman et al. 2023; Trucco et al. 2023), even after the care recipient has died and their care has ended (Mroz et al. 2024).

Mitigation of poor wellbeing is thus essential for caregivers of PWND. The construct of self-compassion has recently emerged as a promising feature to reduce stress and foster wellbeing (Gallego-Alberto et al. 2021; Hlabangana and Hearn 2020). Self-compassion refers to openness to one’s own suffering and experiences, feelings of kindness toward oneself, remaining nonjudgmental when faced with shortfalls, and awareness of a shared human experience (Neff 2003). Self-compassion has been increasingly studied for its potential to mitigate adverse psychosocial outcomes amid life stressors (Barnard and Curry 2011; Bluth and Neff 2018; Brown et al. 2019). Broadly, those who report higher self-compassion also report lower depression and anxiety and higher wellbeing (Goodridge et al. 2021), demonstrating potential for self-compassion as critical mechanism of positive psychological outcomes. In caregivers specifically, mindful self-compassion approaches, that is, self-compassion practices with nonjudgmental attention to the present moment, have been found to reduce stress, anxiety, and emotional distress related to caregiving activities (Hudson et al. 2020). Most popular in recent years is the integration of the concept of self-compassion into interventions that target mindfulness (Barnard and Curry 2011). As the field of self-compassion research grows to envelop populations of caregivers of PWND, a review of research to-date will offer a deeper understanding of the value of caregivers’ self-compassion for navigating caregiving, achieving care goals, and improving wellbeing across and after the caregiving role.

The current study presents a scoping review of literature, mapping the emergence and operationalization of self-compassion in the context of caregiver wellbeing of caregivers of PWND. The research question guiding this review is: How is the concept of self-compassion incorporated into, and characterized within, research on caregiver wellbeing of PWND? Our aims are to understand the scope of peer-reviewed research on self-compassion as it relates to wellbeing in this population, including the measurement tools used to examine self-compassion in the population, and provide guidance for its ideal characterization for future design of potent, effective interventions. This review distinguishes the ways in which self-compassion is used in literature centering caregiver wellbeing, and identifies self-compassion scales used (e.g., existing self-report surveys). Additionally, we describe the scope of the construct of self-compassion in 2 distinct ways: (1) as a primary outcome or marker of wellbeing and (2) as a predictor of other markers of wellbeing (i.e., quality of life, psychological distress, burden, coping), representing a potential mechanism of behavior change (i.e., mediator).

## Methods

Resources outlined in The Cochrane Handbook, such as the Feasible, Interesting, Novel, Ethical, and Relevant (FINER) criteria provided a foundational framework to determine the appropriateness of study type (Higgins et al., 2023), and were used to refine the boundaries of this review. This study is registered with the Open Science Framework (OSF). The Preferred Reporting Items for Systematic Reviews and Meta-analysis extension for Scoping Reviews (PRISMA-ScR) was used as a framework for reporting.

### Rationale

This topic bridges both medical and social literature. Frameworks for performing a scoping study were used from Arksey and O’Malley (2005), founded in the social sciences, and Levac et al. (2010), founded in health research. We chose to conduct a scoping review, rather than a different methodology because the purpose of the review is to map the literature involving self-compassion specifically for caregivers of PWND, rather than respond to a particular question about caregiver self-compassion. Moreover, scoping reviews seek to provide a narrative account of what exists in the literature, rather than a synthesis and assessment of quality of evidence (Arksey and O’Malley 2005). This review provides an innovative yet rigorous framework to blend scientific disciplines in capturing evolving research landscapes.

### Search strategy

The published literature was searched using strategies created by a medical librarian for self-compassion and caregivers. The search was implemented on February 13, 2023, in Embase.com 1947-, PubMed 1946-, and Web of Science 1900-. The search used a combination of standardized terms and keywords without any limits or filters. Keywords and subject headings related to self-compassion and caregiver were identified through preliminary searches. Full search strategies are provided in the Appendix.

### Eligibility

Several criteria were used to select relevant studies. First, most study methodologies were eligible for inclusion: mixed methods, observational, qualitative, intervention including randomized control trials, and quasi-experimental designs. Hypothesis generating literature such as theoretical frameworks and concept papers were also included. Reports and scale validations were excluded. Second, only peer-reviewed articles written in English were included (Pieper and Puljak 2021), to meet research team language capabilities. Third, studies were included when self-compassion was described as part of the caregiver experience either (1) as a measured construct applied by the research team, or (2) as emerging from caregivers’ reflections on their caregiving experiences. Fourth, any health measure captured (i.e., mental, emotional, physical) was included to capture the breadth of wellbeing. Fifth, we focused on adult caregivers of PWND as a population of interest. Articles were excluded if caregivers performed care in any professional capacity (e.g., physicians, nurses, aides, etc.) for individuals to whom they had no pre-existing social relationship, or if the caregiver was a minor. Lastly, care recipients with diagnoses of Alzheimer’s disease, Parkinson’s disease, amyotrophic lateral sclerosis (ALS), prion disease, motor neuron disease, Huntington’s disease, spinal muscular atrophy, and spinocerebellar ataxia were included, as consistent with the molecular medicine classification of the most common NDs (Lamptey et al. 2022).

### Data extraction and management

To remain consistent in the sorting process, the reviewers independently reviewed the same subset of 3 articles to compare sorting results and discuss rationale. Upon agreement and clarity of definitions and operationalization of terms, remaining articles were independently and blindly sorted based on abstract information, using the collaboration platform Rayyan. Any articles that presented a conflict were discussed and the reviewers came to a consensus. All conflicts came to consensus. Data tracking was performed using an excel spreadsheet with article information including author, year of publication, title, study design (i.e., quantitative, qualitative, intervention, or mixed methods), participant information (i.e., country, number of participants, demographics, care recipient conditions), context in which self-compassion was presented, measures or scales used, and study findings. To address our research question, our initial search resulted in a total of 563 articles. Duplicates were removed from Endnote (*n* = 213), bringing the final total to 350 unique citations.

### Data analysis

As outlined in Arksey and O’Malley’s (2005) framework, we identified and selected relevant studies, charted the data, and conducted a descriptive summary analysis to describe the overall pattern of participant characteristics, study types, care recipient conditions, and measurement used for self-compassion. Then, using qualitative content analysis techniques as recommended by Levac et al. (2010), studies were grouped by the characterization and incorporation of self-compassion each study: conceptual integration, emergent theme, associated with other psycho-behavioral outcomes, major outcome. The authors then wrote descriptions of the articles in each group and reviewed those descriptions for accuracy and comprehension. Conceptual integration occurred when self-compassion was used as an idea or a component of wellbeing, but not actually measured by researchers or brought up by participants qualitatively. Self-compassion emerged as a qualitative theme during inductive analysis, revealing that self-compassion is an essential component to navigating and coping in the caregiver experience. It was described as a predictor variable when it was measured as an independent variable in a quantitative study where associations were tested with dependent variables. Finally, self-compassion was described as an outcome variable when it was measured as an endpoint or dependent variable, either predicted by other variables or compared in a pre-posttest design to assess an intervention.

## Results

**Figure 1.**
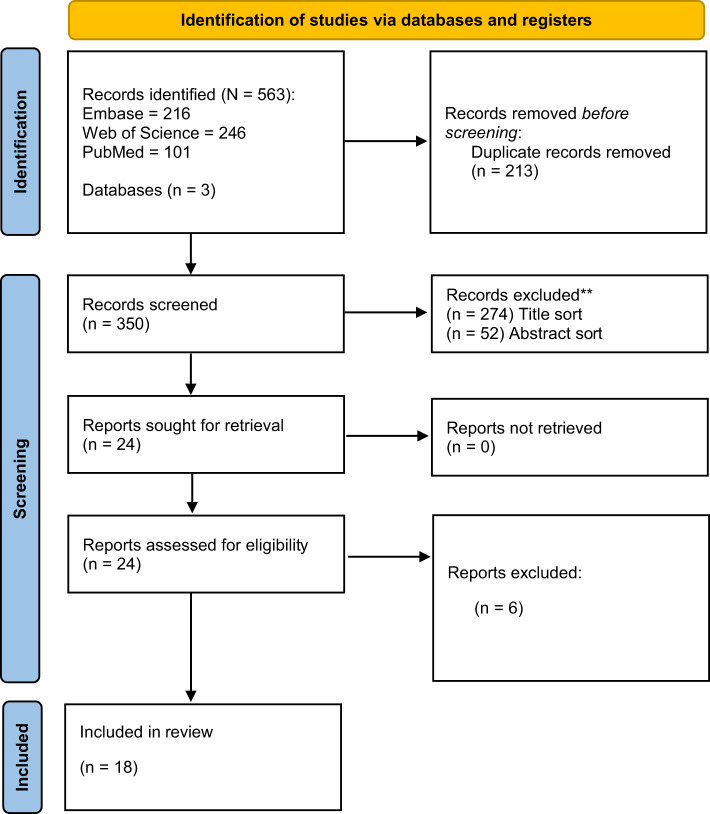
Systematic review flow diagram.

The 350 unique citations were sorted on topic and title and compared against inclusion criteria. Of those, 76 remained to be sorted from abstract content, which was completed by 2 reviewers (CML, SP). An additional 52 articles were eliminated, leaving 24 articles for full text review, all of which were available for retrieval. Reviewers (CML and SP) evenly divided articles, each reading 12 and completing data tracking. After a full text review, 6 articles were excluded, leaving 18 articles included in the review (Figure 1).

**No automation tools were used. *From*: Page MJ, McKenzie JE, Bossuyt PM, Boutron I, Hoffmann TC, Mulrow CD, et al. The PRISMA 2020 statement: An updated guideline for reporting systematic reviews. BMJ 2021;372:n71. doi: 10.1136/bmj.n71. For more information, visit: http://www.prisma-statement.org/.

### Participant characteristics

Trends in study characteristics (i.e., country, number of participants, demographic, care recipient condition) in the reviewed articles are summarized in Table 1. Among those selected, 15 articles (83%) focused only on caregivers. The others were dyadic studies that included measures completed by care recipients as well as caregivers. Sample size ranged from 7 to 141 individuals depending on the study type (e.g., intervention, quantitative, qualitative, and mixed studies). Study participants were mostly middle-aged females. Studies were conducted in the U.S. (Han et al. 2022; Jain et al. 2022; James et al. 2021; Spigelmyer et al. 2023; Yang et al. 2022), Canada (Goodridge et al. 2021; Zarei et al. 2022), the United Kingdom (Contreras et al. 2021; Diggory and Reeves 2022; Hlabangana and Hearn 2020; Jones et al. 2019; Lloyd et al. 2019), Europe (Berk et al. 2019; Giménez-Llort et al. 2021), Israel (Tolmacz and Pardess 2022), Australia (Murfield et al. 2020b), Brazil (Danucalov et al. 2017), and Japan (Tamura et al. 2023).

**Table 1.** Self-compassion in caregivers of persons living with neurodegenerative diseases

Author, year	Country	Study type	Number of caregivers	Participant demographics (age, self-identified gender, race)	Care recipient conditions	Self-compassion measurement	Operationalization of self-compassion and main results
**Self-compassion conceptually integrated**							
**Goodridge et al. (2021)**	CA	Intervention	29	Age (*M* = 59.6), Female (*N* = 26), Race/Ethnicity not indicated	Dementia	None	Self-compassion was a concept underpinning the development of a mindfulness intervention. There was not pre-post change in reported caregiver burden, but there was pre-post improvement in reported emotional-wellbeing and decreased emotion-based coping.
**Diggory and Reeves (2022)**	UK	Mixed methods	9	Age (*M* = 58), Female (*N* = 9, 100%), Race (White British, *N* = 9)	A variety of illnesses including motor neuron disease, end stage cancer, and dementia	None	Self-compassion is embedded in a self-care intervention. Participants described self-compassion as showing up in 3 ways: being your own best friend, permission to be kind to oneself, and gains of making participants feel better.
**Tolmacz and Pardess (2022)**	Israel	Conceptual framework	None	None	Dementia	None	Self-compassion is used in the development of a theoretical framework for the wellbeing of caregivers of those with dementia.
**Self-compassion as emergent theme**							
**Jones et al. (2019)**	UK	Qualitative	13	Age (*M* = 65.7) Self-identified gender not indicated, Race/Ethnicity not indicated	Dementia	None	Self-compassion emerges from interviews assessing caregiver perceptions of resilience. Participants describe self-compassion as a buffer to the stress of caregiving. Findings highlight resilient caregivers prioritize self‐compassion as key to resilience.
**Han et al. (2022)**	US	Intervention	7	Age (*M* = 57.3),Female (*N* = 6), Race (Non-Hispanic White [*N* = 5], Black or African American [*N* = 1], Native American [*N* = 1])	Dementia	None	Self-compassion and its components are identified by caregivers in post-intervention interviews to increase psychological flexibility.
**Yang et al. (2022)**	US	Intervention	11	Age (range: 38–65+), Female (*N* = 9), Race (Caucasian [*N* = 7], African American [*N* = 2], Hispanic [*N* = 2])	Dementia	None	Self-compassion emerged organically as a psychological mediator in caregiver interviews after completing a mentalizing imagery therapy (MIT) program. Participants described increased self-compassion as effecting wellbeing and enabling coping with challenging situations.
**Self-compassion as associated with other psycho-behavioral outcomes**							
**Lloyd et al. (2019)**	UK	Quantitative hypothesis generating	73	Age (*M* = 67.2), Female (*N* = 54), Race (White British [*N* = 69], White Non-European [*N* = 2], other [*N* = 2])	Dementia	Self-Compassion Scale Short Form	Higher self-compassion was found to be predictive of decreased caregiver burden, decreased poor coping strategies, and increased emotion-focused coping strategies. Low self-compassion led to poor coping strategies, which mediates caregiver burden.
**Hlabangana & Hearn (2020)**	UK	Quantitative hypothesis generating	57	Age (*M* = 58.2), Female (*N* = 41), Race (Asian/British Asian [*N* = 4], Black/African/Caribbean/Black British [*N* = 3], White British/Caucasian [*N* = 43], Mixed/Multiple ethnic groups [*N* = 3])	Spinal cord injury, dementia, cancer, multiple Sclerosis, and other chronic disease	Self-Compassion Scale Short Form;	Higher self-compassion is associated with higher quality of life, except physical quality of life. Higher self-compassion is also associated with lower depression symptoms.
**Contreras et al. (2021)**	UK	Quantitative hypothesis generating	91	Age (*M* = 69.5),Female (*N* = 61), Race/Ethnicity not indicated	Dementia	Self-Compassion Scale Short Form;	Self-compassion was not found to be predictive of caregiver quality of life.
**Giménez-Llort et al. (2021)**	Spain	Quantitative hypothesis generating	24	Age (range: 18–54),Female (*N* = 16), Race/Ethnicity not indicated	Multiple sclerosis	Self-Compassion Scale Short Form; Compassion Scale (CS)	Self-compassion was not found to be predictive of overall health and fatigue in caregivers.
**Murfield et al. (2020b)**	AU	Quantitative hypothesis generating	141	Age (*M* = 59.6),Female (*N* = 121), Race/Ethnicity not indicated	Dementia, multiple sclerosis, cardiovascular, cancer, Parkinson’s disease	Compassionate Engagement and Action Scales (CEAS)	Higher self-compassion is associated with lower depressive symptoms, anxiety, and stress. Higher self-compassion is also associated with emotion- and problem-focused coping.
**Spigelmyer et al. (2023)**	US	Intervention study	24	Age (*M* = 66.2, *SD* = 11.5), Female (*N* = 20, 83%), Race (American European [*N* = 24, 100%])	Dementia	Self-Compassion Scale	A Mindful Self-Compassion (MSC) intervention decreased caregiving fatigue and perceived stress.
**Self-compassion as the study’s major outcome**							
**Danucalov et al. (2017)**	Brazil	Intervention	46	Control: Age (*M* = 53.4, *SD* = 8.2), Female (*N* = 19, 90%) Intervention: Age (*M* = 55.5, *SD* = 8.1),Female (*N* = 22, 88%) Race/Ethnicity not indicated	Dementia (Alzheimer’s disease)	Self-Compassion Scale	An intervention using yoga increased self-compassion outcome measures.
**Berk et al. (2019)**	Netherlands	Intervention	14	Age (*M* = 70.75, *SD* = 8.14),Female (*N* = 5, 71%) Race/Ethnicity not indicated	Dementia	Self-Compassion Questionnaire Short Form	A Mindfulness-Based Stress Reduction (MBSR) intervention made little difference in caregiver self-compassion, but a moderate difference in overall caregiver quality of life.
**James et al. (2021)**	US	Intervention	10	Age (*M* = 60.3, *SD* = 7.4), Female (*N* = 14, 73.7%) Race (White [*N* = 17], Black/African American [*N* = 1], American Indian/Alaska Native [*N* = 1])	Dementia (Alzheimer’s disease)	Self-Compassion Scale Short-Form	An intervention using heart-focused breath was not significant self-compassion outcome measures, between control and intervention group.
**Jain et al. (2022)**	US	Intervention	46	Control: Age (*M* = 61, *SD* = 8.4), Female (*N* = 21, 87.5%),Race (White/Caucasian [*N* = 17, 70.8%], Asian [*N* = 4, 16.7%], Black/African American [*N* = 3, 12.5%]) Intervention: Age (*M* = 66.1, *SD* = 9.8), Female (*N* = 16, 72.7%)Race (White/Caucasian [N = 17, 77.3%], Asian [*N* = 3, 13.6%], Black/African American [*N* = 0, 0%], more than 1 race [*N* = 2, 9.1%])	Dementia	Self-Compassion Scale Short-Form	An intervention using mentalizing imagery therapy (MIT) increased self-compassion outcome measures.
**Zarei et al. (2022)**	CA	Intervention	26	Control: Age (*M* = 63, *SD* = 15), Female (*N* = 10, 83%), Intervention: Age (*M* = 58, *SD* = 11),Female (*N* = 13, 92%) Race/Ethnicity not indicated	Dementia	Self-Compassion Scale	An intervention using tele-mindfulness increased self-compassion outcome measures.
**Tamura et al. (2023)**	Japan	Intervention study	64	Control: Age (*M* = 66.3, *SD* = 12.1), Female (*N* = 22, 68.8%) Intervention: Age (*M* = 63.5, *SD* = 12.7),Female (*N* = 23, 74.2%) Race/Ethnicity not indicated	Dementia	The Japanese version of the Self-Compassionate Reactions Inventory (SCRI-J)	A cognitive behavioral therapy (CBT) program did not impact self-compassion in caregivers.

*Note:* Number of participants refers to the participating caregivers only; information on included care recipients is not provided.

Most study participants were White with few articles including specific racial or ethnic backgrounds such as Black or African American, Hispanic, Native American, Asian, or mixed/multiple ethnic groups (Han et al. 2022; Jain et al. 2022; Yang et al. 2022). The diagnoses of care recipients included Alzheimer’s disease and related dementias in most studies (95%). In 4 (21%) articles, PWND such as multiple sclerosis (Giménez-Llort et al. 2021; Hlabangana and Hearn 2020; Murfield et al. 2020b), Parkinson’s disease (Murfield et al. 2020b), and motor neuron disease (Diggory and Reeves 2022) were also included, as well as individuals living with other chronic, progressive illness including cancer (Diggory and Reeves 2022; Hlabangana and Hearn 2020; Murfield et al. 2020b), and spinal cord injury (Hlabangana and Hearn 2020).

### Inclusion of self-compassion as a research construct

Self-compassion was addressed in 4 ways across studies: (1) applied as conceptual integration (3 articles, 16%), (2) emergent theme by the participant narrative (3 articles, 16%), (3) as a predictor (5 articles, 27%), and (4) as a major outcome (7 articles, 39%) of the study. In our review, we focus on main findings from these studies regarding caregiver self-compassion.

#### Self-compassion conceptually integrated

Conceptual integration is used here as the merging of 2 or more concepts into a cohesive scheme. We found self-compassion merged with several different areas of research such as an underpinning of the larger research framework (Goodridge et al. 2021; Spigelmyer et al. 2023), embedded in an intervention where feedback about experiencing self-compassion was elicited via participant narrative (Diggory and Reeves 2022), and as a concept to create a larger theory of caregiver wellbeing (Tolmacz and Pardess 2022). In 2 intervention studies (Diggory and Reeves 2022; Goodridge et al. 2021), education about self-compassion was presented to participants as part of mindfulness training programs. In a pre-post mindfulness-based self-compassion intervention (Goodridge et al. 2021), there was no notable change in caregiver burden (e.g., the Burden Scale for Family Caregiving), but increases in emotional wellbeing (e.g., World-Health Organizations-5) and decreases in emotion-based coping (e.g., Brief COPE). Diggory and Reeves (2022) findings highlight that a brief self-care intervention using a self-compassion framework can have a positive impact on caregivers’ reported wellbeing through helping caregivers develop a kinder internal orientation and permission to practice intentional self-care.

Self-compassion was also included as a core component of a conceptual model of caregiver wellbeing for caregivers of those living with dementia, (Tolmacz and Pardess 2022). This model asserts that caregiver wellbeing is predicated on caregivers’ ability to balance care and compassion for others (e.g., the care recipient living with dementia) and care and compassion for self. The model suggests that the balance of self-compassion is inherently context-dependent and becomes more challenging to maintain as disease progresses. According to the model, social norms may reinforce the imbalance through negative attitudes, bias, and stereotypes (e.g., ageism), further isolating caregivers. The authors posit that the cultivation of self-compassion and integration of compassion into caregiver interventions restores balance not only on the individual level of the caregiver, but beyond to the family level, where extended family provides support as well (Tolmacz and Pardess 2022).

#### Self-compassion as emergent theme from participants

In 1 qualitative (Jones et al. 2019) and 2 intervention studies (Han et al. 2022; Yang et al. 2022), self-compassion arose from data as an emergent theme. Jones (2019) describes caregiver’s propensity for being self‐compassionate in terms of accepting personal limitations and adapting expectations. In intervention studies, self-compassion was an area commonly mentioned by participants as newfound ability to find comfort, aspects of forgiveness, and empathy while struggling with depression (Yang et al. 2022). In a similar intervention study (Han et al. 2022), no specific measure of self-compassion was used, rather qualitative themes emerged from post-intervention caregiver interviews, where caregivers reportedly recognized the need to be more self-compassionate. Authors note that these findings are congruent with specific components of self-compassion (e.g., shared humanity, mindfulness) (Bluth and Neff 2018) and were captured by the study’s subtheme of *being more patient with relatives with dementia*.

#### Self-compassion as a predictor of outcomes

Self-compassion was used as a predictor of main study outcomes in 5 quantitative studies (Contreras et al. 2021; Giménez-Llort et al. 2021; Hlabangana and Hearn 2020; Lloyd et al. 2019; Murfield et al. 2020b). In 3 studies, self-compassion was negatively associated with psychological distress (e.g., caregiver burden) and dysfunctional coping strategies (Hlabangana and Hearn 2020; Lloyd et al. 2019; Murfield et al. 2020b). Low self-compassion was found to be related to high caregiver burden, poor coping strategies, and poor emotion-focused coping (Lloyd et al. 2019). Also, dysfunctional coping strategies were found to mediate the relationship between self-compassion and caregiver burden (Lloyd et al. 2019). Hlabangana and Hearn (2020)included the number of hours spent caregiving *(M* = 8.90, SD = 6.40) and determined that more hours providing care and lower self-compassion are predictive of higher depression. Here again, self-compassion was negatively associated with burden, dysfunctional coping, and emotion-focused coping. In addition, Murfield et al. (2020b) found that higher self-compassion was associated with lower emotion regulation difficulties and psychological distress and associated with better adaptive coping strategies. Following engagement in the Mindfulness Self-Compassion program conducted by Spigelmyer et al. (2023), coping scores (i.e., Ways of Coping) indicated some positive change in the ways in which caregivers think about stressful situations.

Alternatively, Contreras et al. (2021) found self-compassion was not a predictor of caregiver wellbeing. Moreover, self-compassion was found not to be a predictive factor in physical and mental health in caregivers when examining the secondary impacts of the COVID-19 pandemic (Giménez-Llort et al. 2021).

#### Self-compassion as an outcome

Interventions studied psychological distress (e.g., stress and anxiety) and used integrative approaches to intervene (e.g., mindfulness, yoga, breath training) (Berk et al. 2019; Jain et al. 2022; Spigelmyer et al. 2023; Tamura et al. 2023; Zarei et al. 2022). Most intervention studies found improvement in self-compassion post-intervention (Berk et al. 2019; Jain et al. 2022; Zarei et al. 2022). Berk et al. (2019) conducted a 2-week mindfulness-based intervention and found improvement in self-compassion scores among caregivers of persons living with dementia. Zarei et al. (2022) conducted a tele-mindfulness intervention and found improvement in self-compassion, indicating the effectiveness of the intervention. Similar to mindfulness-based interventions, Jain et al. (2022) conducted a mentalizing imagery therapy and found that the intervention group experienced improved self-compassion and other positive psychological traits (e.g., mindfulness, wellbeing, acceptance). Specifically, self-compassion measures were higher in the post mentalizing imagery therapy group as compared to the control group which attended a standard support group (Jain et al. 2022). In contrast, Tamura et al. (2023) completed a group multi-component program based on cognitive behavioral therapy and positive psychology but could not identify difference in changes of self-compassion within intervention group, nor between intervention and control groups.

Two studies used physiological interventions to assess self-compassion (Danucalov et al. 2017; James et al. 2021) but had contradicting findings. No improvement in self-compassion occurred after a virtual heart-focused breathing program (James et al. 2021), however, in an intervention using yoga and compassion meditation was shown to be beneficial in improving self-compassion (Danucalov et al. 2017).

### Measures of self-compassion

Self-compassion was a measured outcome in 7 intervention studies (Berk et al. 2019; Danucalov et al. 2017; Jain et al. 2022; James et al. 2021; Spigelmyer et al. 2023; Tamura et al. 2022; Zarei et al. 2022). Of the studies that used a questionnaire-style self-report measure of self-compassion (66%, *N* = 12), 58% (*N* = 7) chose the Self-Compassion Scale – Short Form (SCS-SF). The full version of the SCS (Raes et al. 2011) was used in 25% (*N* = 3) of studies. Murfield and colleagues (2020) study used a sub scale of Compassionate Engagement and Action Scales (CEAS) (Gilbert et al., 2017)to measure compassion experienced for others, from others, and self-compassion. Tamura et al. (2023) utilized the Self-Compassionate Reactions Inventory – Japan (SCRI-J). This scale measures the level of self-compassion based on the degree to which people choose self-compassionate reactions to 8 hypothetical hardships (Miyagawa and Taniguchi, 2016).

## Discussion

Self-compassion was operationalized in several ways within the context of promoting psychological wellbeing for caregivers of PWND: it manifested as a component of integrated models of caregiver wellbeing, emerged as a core aspect of wellbeing through interviews with caregivers, was positioned as a predictor of common metrics of caregiver wellbeing (e.g., burden), and tested as an outcome of caregiver behavioral interventions. The literature varies in its application of self-compassion and description of how it may be most beneficial to caregiver wellbeing. Some studies integrate self-compassion as a component of mindfulness, consistent with the identified components (e.g., non-judgment) of both self-compassion and mindfulness (Bluth and Neff 2018). However, when using self-compassion as an integrated concept, studies did not measure self-compassion itself, rather imbedded it in frameworks or models to discuss other outcomes of interest (e.g., caregiver wellbeing, burden, kindness). Lack of unified definition and positioning of self-compassion in caregiver research can create inconsistencies in the literature and barriers to understanding the conceptualization of self-compassion in caregiver wellbeing.

When measured, the same tool is often used, the SCS either in long form or more often in short form. This may be due to limited measurement tools available fit to populations of caregivers of PWND (Murfield et al. 2020a). Given that the SCS or SCS-SF is frequently used within this caregiver population, validation of fit may be needed to ensure accuracy of measurement. Should evidence support adaptation, the SCS has already evolved into a validated short form measure and has been adapted for youth populations (Neff et al. 2021), showing promise in its ability for adaptation to other groups. Future researchers are well-positioned to continue such adaptations and validations.

Of the studies that included the caregiver-care recipient dyad, none delved into the concept of self-compassion within the type of relationship comprising the dyad. For example, caregiver self-compassion may appear or function differently between spouses rather than a parent and adult child pairing. Few of the studies included in this review assess the length of time as a caregiver, time spent caregiving, or additional caregiving supports used, where previous evidence finds associations between time spent caregiving and psychological wellbeing (Contreras et al. 2021). There is limited understanding of the external caregiving contexts that promote or inhibit this internal psychological process for this specific population of caregivers. There is also limited exploration of actor-partner interdependence (Allore et al. 2020), where, for example, care recipient features may influence caregiver self-compassion, or vice versa.

### Envisioning self-compassion within the landscape of intervention development

One important takeaway from this scoping review is that our understanding of self-compassion within frames of caregiving for PWND is still in its infancy, with the oldest study in this review from 2017 (Danucalov et al. 2017). Many studies inductively identified self-compassion as a central aspect of caregiver wellbeing, underscoring the value of addressing self-compassion in interventions. The scale most often referenced in the studies within this review was originally developed in 2003 (Neff 2003), however the study of self-compassion and use of measures is notably more recent. To strengthen understanding of these measurement tools used to examine self-compassion in caregiver wellbeing, future reviews should examine the validity of measurements used as well as the quality of intervention studies performed.

In future studies, it is essential that the conceptualization of self-compassion be consistent in its operationalization. But what is the best way to conceptualize self-compassion within the landscape of caregiver wellbeing, and how should researchers translate concepts to building effective and potent interventions? The National Institutes of Health (NIH) has recently urged researchers to frame intervention development by defining and measuring mechanisms that underlie behavioral patterns that are targets of change (Nielsen et al. 2018). Researchers argue that the process of developing interventions, particularly complex or multi-component interventions targeting broad outcomes (e.g., caregiving patterns of stress, wellbeing, burden) is enhanced when researchers form and test hypotheses about why behaviors exist and how they are changed through intervention practices. Improving science by identifying and testing mechanisms can happen at all intervention development stages, but this is difficult to do when there is conflicting guidance about how to position constructs when forming hypotheses about behaviors and behavior change, as is the case of self-compassion as a construct.

In the case of self-compassion in this caregiver population, our review shows that existing studies have positioned self-compassion as both a potential mechanism of change (i.e., we can teach self-compassion skills, measure self-compassion changes, and determine how improvements in self-compassion are associated with improved clinical endpoints) and as an outcome variable (self-compassion is itself a clinical endpoint, as a marker of or aspect of caregivers’ wellbeing). Interestingly, observational research often positions self-compassion as a predictor variable (Giménez-Llort et al. 2021; Lloyd et al. 2019; Murfield et al. 2020b), and therefore a potential mechanism of change once embedded in intervention trials. Existing intervention research positions self-compassion as an outcome (Berk et al. 2019; Danucalov et al. 2017; Jain et al. 2022; James et al. 2021; Tamura et al. 2022; Zarei et al. 2022), thus implying that self-compassion should stand as a clinical endpoint, particularly following mindfulness or related behavioral interventions. This spectrum may unintentionally obscure the best positioning of self-compassion in conceptual models or research designs.

Through our observations of existing research and caregiver-defined social and emotional needs (Zhang and Bennett 2024)we encourage researchers to focus on self-compassion as a potentially critical *mechanism of change* for caregivers of PWND. In this caregiving population specifically, self-compassion may serve as a necessary defense against common feelings of guilt, self-doubt, frustration, including in contexts when caregivers are ashamed of their perceived limitations, expressions of negative emotions, or changing mental representations of their relationship with their care recipient (Gallego-Alberto et al. 2021). By doing so, self-compassion forms the link between negative caregiver outcomes and the origins of those outcomes (e.g., high stress as a result of intense feelings of guilt). This assertion could provide some clarity in the development of conceptual models that underpin interventions to increase caregiver wellbeing and reduce stress and psychological problems (i.e., our proposed clinical endpoints). We recognize that the existing interventions we reviewed in this article, those that target self-compassion as a primary outcome, may be quite useful for caregivers, but that expanded testing of those interventions may benefit from measurement of distal outcomes (e.g., reduced stress) as well as more proximal measurement of caregiver self-compassion. As research teams move these interventions along the NIH stages of development (Onken, 2014), incorporating behavior change science in that work will help revise or accentuate the assertions we make here.

### Future directions: Deepening understanding of caregiver self-compassion across and populations

The classification of ND is broad, but include a variety of distinct disease types, each with unique characteristics and care needs. Heterogeneity among care recipient diagnosis was not represented in this data. In these studies, ND was overwhelmingly represented by samples of persons living with Alzheimer’s disease or related dementias. Over half of all caregivers in the US provide support to the over 11 million adults currently living with Alzheimer’s disease or related dementias (AARP 2020), which may partially explain researchers’ focus to-date on caregivers in this population. This focus on caregivers of persons living with dementia, however, overshadows entire populations of caregivers for those with ND outside of dementia contexts. Other NDs in studies in this review were multiple sclerosis, broad motor neuron disease, and Parkinson’s disease. These are some of the most common NDs (Lamptey et al. 2022), however ALS, Huntington’s disease, prion disease, and spinocerebellar ataxia are also common NDs (Lamptey et al. 2022) but were not represented in the care recipient samples identified through our review. Future research should explore the nuances of self-compassion when caregiving for persons living with other NDs, as symptomology, disease trajectory, and care provision vary across these caregiving contexts. In addition, given the increasing functional debility of persons living with most types of ND, caregiving intensity increases in kind, yet this unique feature of caregiving remains unexplored in the literature. Discussions in gerontological literature already lean in the direction of exploring caregiving at a more diagnosis-specific level (Adelman et al. 2014).

Caregivers, across cultures, in this review were overwhelmingly female. Literature centering caregiving within feminist and social justice frameworks note the evolving cultural expectations placed on women as well as shifts in power infused into the caregiving experience. This contributes to a gender imbalance in the participation of caregiving (Mackinnon 2009). Some research highlights an intersection between mindfulness and self-compassion as a means by which women may reclaim connection to the self after caring for someone else (Crowder 2016), thereby taking back their power. The question of imbalance remains: although the data may accurately represent the proportion of women acting as caregivers, there is limited understanding of the gender differences in caregiver burden and wellbeing (Xiong et al. 2020), and how mindfulness and self-compassion may, or may not, be beneficial for a male caregiver. Caregivers represented in this review were also predominantly White, which is not representative of the caregiving population as a whole (Garcia et al. 2019; Liu et al. 2020). This suggests that conceptualizations and interventions are normed on the populations from which they are studied, and current interventions are disproportionately irrelevant to non-White caregivers. Shifting frameworks to inclusive (e.g., sociocultural and intersectional) theoretical perspectives and intentional recruitment of participants is necessary for future work (Dilworth-Anderson et al. 2020). Self-compassion aligns comfortably with diversity and inclusion areas of opportunity, as one of the foundational elements is shared humanity (Barnard and Curry 2011). The act of caregiving inherently demonstrates this foundational component of self-compassion as seeing one’s experience as a part of the larger human experience by realizing that suffering is shared (Neff 2003). In doing so, self-compassion fosters empathy and non-judgement, reducing bias and contributing to inclusivity.

### Study limitations

Although a scoping review framework was used (Arskey and O’Malley 2005; Levac et al. 2010), given the heterogeneity of study types and context in which self-compassion is used in the literature, a quality assessment tool was not used. As such, there is unknown bias present in the studies included in review, possibly biasing the findings we presented. Within the framework employed, Arskey and O’Malley (2005) recommend optional consultation of investors or interested parties as a way to ensure accuracy of findings. Consultation was not used which may lend to unidentified gaps in our work. As stated in the aims of the study, only peer-reviewed research was included, omitting literature that is not peer reviewed, in-progress, or not published. Given that this is a budding area of research, information from upcoming publications could have been missed. Additionally, literature published in English was eligible for inclusion, which may cause potential bias in the scope of work reviewed here.

## Conclusion

Our scoping review mapped the inclusion of self-compassion in the context of caregiver wellbeing of caregivers of PWND. Diagnoses like dementia, Parkinson’s disease, ALS, and others have a dramatic and lengthy trajectory with increasingly complex care needs. Self-compassion has been incorporated into larger intervention frameworks, is often measured using a tool designed originally to address mindfulness, and is conceptualized as both a predictor or mechanism of change and an outcome or wellbeing endpoint (Goodridge et al. 2021; Han et al. 2022; Hudson et al. 2020; Sanchez-Perez et al. 2022; Uneno et al. 2022). Clarity of concept and proof of impact are critical in employing evidence-based care (Barnard and Curry 2011). Additionally, norming and adaptation of measurement is needed, as the self-compassion tools available have yet to be normed or adapted for specific caregiver populations. Future work may specify the role of self-compassion in the caregiver experience, as well as its function as a mechanism of change to foster wellbeing.

## Supporting information

Lero et al. supplementary material 1 Lero et al. supplementary material

Lero et al. supplementary material 2 Lero et al. supplementary material

## Supplementary material

The supplementary material for this article can be found at https://doi.org/.10.1017/S1478951524001639.
