# Supplementary material for: Mapping the evidence of self-compassion in caregiver wellbeing for caregivers of persons with neurodegenerative disease: A scoping review
Source: Palliat Support Care. 2025 Jan 21;23:e38. doi: 10.1017/S1478951524001639 (PMC12501646; doi:10.1017/S1478951524001639)
Supplement: Lero et al. supplementary material 2 — Lero et al. supplementary material [file S1478951524001639sup002.docx]

**Appendix A: Full Search Strategies**

Total: 563
Duplicates: 213
Unique: 350

Embase.com
216 results on 2/13/23

1. 'self compassion'/exp OR selfcompassion:ti,ab,kw,de OR 'self compassion':ti,ab,kw,de OR ((compassion* NEAR/2 (self OR oneself)):ti,ab)

2. 'caregiver'/exp OR 'care giver':ti,ab,kw,de OR caregiver:ti,ab,kw,de OR 'caregivers':ti,ab,kw,de OR 'carer':ti,ab,kw,de OR caregiving:ti,ab,kw,de OR 'carers':ti,ab,kw,de OR 'family caregiver':ti,ab,kw,de OR 'family caregivers':ti,ab,kw,de OR spouse:ti,ab,kw,de OR parent:ti,ab,kw,de

3. 1 AND 2

Web of Science
246 results on 2/13/23

**1. TS= ("selfcompassion" OR "self-compassion" OR "self compassion" OR (compassion* NEAR/3 (self OR oneself)))**

2. TS= ( "care-giver" OR "care giver" OR "caregiver" OR "caregivers" OR "carer" OR "caregiving" OR "carers" OR "family caregiver" OR "family caregivers" OR "spouse" OR "parent" OR “sibling” OR “siblings”)

3. 1 AND 2

PubMed
101 results on 2/13/23

("Caregivers"[Mesh] OR care giver[TIAB] OR caregiver[TIAB] OR caregivers[TIAB] OR carer[TIAB] OR caregiving[TIAB] OR carers[TIAB] OR spouse[TIAB] OR spouses[tiab] OR sibling[tiab] OR siblings[tiab]) AND ("Self-Compassion"[Mesh] OR selfcompassion[tiab] OR self-compassion[tiab])
